# Objective classification of different head and neck positions and their influence on the radiographic pharyngeal diameter in sport horses

**DOI:** 10.1186/1746-6148-10-118

**Published:** 2014-05-23

**Authors:** Li-mei Go, Ann Kristin Barton, Bernhard Ohnesorge

**Affiliations:** 1 Clinic for Horses, University of Veterinary Medicine Hannover, Foundation, Bünteweg 9, Hannover D-30559, Germany; 2 Equine Clinic, Department of Veterinary Medicine of Freie Universität Berlin, Oertzenweg 19 b, Berlin D-14163, Germany

**Keywords:** Horse, Equine pharynx, Head and neck position, Pharyngeal diameter, Upper respiratory tract

## Abstract

**Background:**

Various head and neck positions in sport horses are significant as they can interfere with upper airway flow mechanics during exercise. Until now, research has focused on subjectively described head and neck positions. The objective of this study was to develop an objective, reproducible method for quantifying head and neck positions accurately.

**Results:**

Determining the angle between the ridge of the nose and the horizontal plane (ground angle) together with the angle between the ridge of nose and the line connecting the neck and the withers (withers angle) has provided values that allow precise identification of three preselected head and neck positions for performing sport horses. The pharyngeal diameter, determined on lateral radiographs of 35 horses, differed significantly between the established flexed position and the remaining two head and neck positions (extended and neutral). There was a significant correlation between the pharyngeal diameter and the ground angle (Spearman’s rank correlation coefficient −0.769, p < 0.01) as well as between the pharyngeal diameter and the withers angle (Spearman’s rank correlation coefficient 0.774, p < 0.01).

**Conclusion:**

The combination of the ground angle and the withers angle is a suitable tool for evaluating and distinguishing frequently used head and neck positions in sport horses. The ground angle and the withers angle show significant correlation with the measured pharyngeal diameter in resting horses. Hence, these angles provide an appropriate method for assessing the degree of head and neck flexion. Further research is required to examine the influence of increasing head and neck flexion and the related pharyngeal diameter on upper airway function in exercising horses.

## Background

During the last few years, various head and neck positions in sport horses have sparked considerable discussion in the equine community. Particularly, extreme flexion of the poll and neck is suspected to cause pathology in horses [1-3]. Several papers have been published about the influence of head and neck positions on locomotion. Other reports suggest that head and neck flexion can induce or deteriorate dynamic upper respiratory tract collapse in exercising horses [4-10]. The effect of head and neck positions on the welfare of sport horses has also been evaluated [11,12]. In most of these studies, head and neck positions were judged only subjectively—by description. To achieve uniformity, a number of authors have used the classification of head and neck positions described by Gómez-Álvarez et al. [13]. These six predetermined head and neck positions were also defined by subjective attributes. Some authors have attempted to standardize head and neck positions using a protractor to measure the angle between the mandible and the ventral neck line [2] or between the intermandibular space and the ventral neck line [14]. In a recent study, four angles and two distances were measured to quantify head and neck positions in walking horses [15]. For the measurements, the authors glued reflective markers on anatomical landmarks and obtained individual frames from lateral videos of the horses. This technique has been used for research only and is too complex and time-consuming for routine examinations.

The aim of the present study was to find an easy, reproducible method for categorizing head and neck positions objectively. We began by introducing two newly defined angles (ground and withers angles). Using these angles, we attempted to quantify objectively the head and neck position of horses shown in pictures taken of them during exercise. In the second part of the study, the pharyngeal diameters of horses with various head and neck positions at rest were calculated on the basis of lateral radiographs of the head. The ground and withers angles were determined simultaneously. Subsequently, we evaluated the causal relation between the angles and the pharyngeal diameter to determine if the angles could be used for estimating the influence of the various head and neck positions on the upper airway.

## Methods

A total of 35 German warmblood horses (one stallion, 23 geldings, 11 mares) with no cardiovascular or musculoskeletal diseases participated in the study. The population evaluated in this study was selected randomly. The horses’ usual working disciplines included dressage (*n* = 9), show jumping (*n* = 13), and pleasure riding (*n* = 13). All of the horses underwent a performance test, and lateral radiographs of the head were obtained at rest. The examinations were carried out at the Clinic for Horses, University of Veterinary Medicine Hannover, Germany. All research carried out on horses were registered and permitted (reference number Az 10A 051) by the Lower Saxonian State Office for Consumer Protection and Food Safety (LAVES, Oldenburg, Germany).

For the exercise test, the horses were ridden at trot and canter in each of the specific head and neck positions. The riders were asked to present their horses unrestrained (without contact between the rider’s hand and the bit), with elevated neck (with the poll being the highest point and the ridge of the nose slightly in front of vertical), and in hyperflexion (with the ridge of the nose behind vertical). All head and neck positions were achieved without the use of auxiliary reins. Each horse was ridden according to its individual level of training so it would perform willingly without resistance to the rider. Occurrence of abnormal respiratory noises, times of appearance, as well as the characteristics and alterations in dependence upon the head and neck positions were noted. Videos of each combination of gait and head and neck position were recorded.

**Figure 1:**
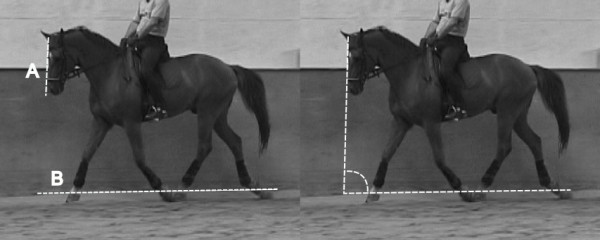
**Ground angle.** The ground angle (at right) was defined as the angle between the ridge of the nose (at left, line A) and the horizontal ground plane (at left, line B).

**Figure 2:**
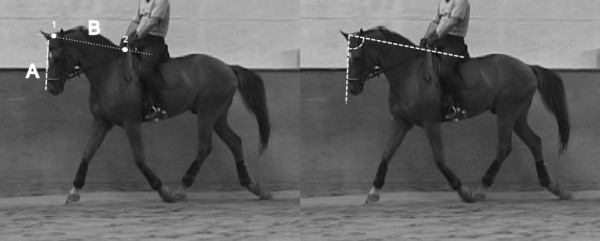
**Withers angle.** The withers angle (at right) was defined as the angle between the ridge of the nose (at left, line A) and the line (at left, line B) connecting the neck (point 1) and the withers (point 2).

The videos were obtained with a digital camera (Sony DCRVX 2000; Sony, Minato, Tokyo, Japan) placed at the middle of a long side of an indoor arena. The horses were filmed while performing on the opposite long side. Freeze frames were captured from these videos using a video-editing program (EDIUS 5.5; Grass Valley, San Francisco, CA, USA). To avoid parallax errors, freeze frames were chosen at the point when the horse was perpendicular to the observation line of the video camera. Also, the horse’s outer front leg was vertical to the ground, or all four feet were off the ground.Based on these freeze frames, the head and neck positions were assessed subjectively and objectively. For the subjective evaluation, the head and neck positions shown on the freeze frames were checked by one of the authors for correct interpretation. Head and neck position was rated as correct if it could be classified in one of the positions (unrestrained, elevated neck, hyperflexion) as defined above. Frozen images from horses that could not be classified in one of these three positions were excluded from further analysis. For objective quantification of the head and neck positions, two newly defined angles (ground and withers angles) were measured using the selected freeze frames. For this purpose, a custom-made addition to the graphic software program (ImageJ 1.45e; rsbweb.nih.gov/ij/) was coded. The ground angle (GA) (Figure 1) was defined as the angle between the ridge of the nose and the horizontal ground plane. The withers angle (WA) (Figure 2) was defined as the angle between the ridge of the nose and the line connecting the neck and the withers.

**Figure 3:**
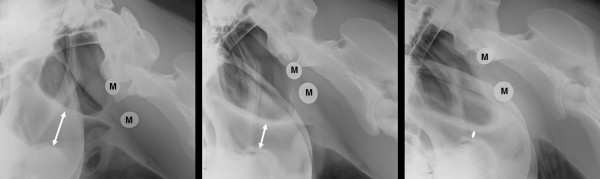
**Lateral radiographs of the head.** Lateral radiographs of the head were obtained in three head and neck positions. Left: extended head and neck position. Middle: neutral head and neck position. Right: flexed head and neck position. The pharyngeal diameter was defined as the shortest distance between the epiglottis and the roof of the pharynx (double-headed arrow). The metallic markers (M) were used to calculate the amplification factor as described by Cehak et al. [16].

For the radiographic examination, all horses were sedated with intravenous detomidine hydrochloride 0.115–0.03 mg/kg (Cepesedan® RP; CP-Pharma GmbH, Burgdorf, Germany) and intravenous butorphanol tartrate 0.1 mg/kg (Alvegesic® PH; CP-Pharma GmbH). Simultaneous with the radiographic examination, lateral photographs were taken of the horses’ head and neck positions. The radiographs were obtained by computed radiography (GIERTH HF 1000; Gierth X-Ray International GmbH, Riesa, Germany ) with standardized settings (80 KV, 166 mA, 0.12 s) without a bucky grid.Lateral radiographs of the head were obtained in three head and neck positions (neutral, extended, flexed) (Figure 3). During the examination, all horses wore a halter with no metal fittings. Two metallic markers with a defined length were attached to either side of the horse’s head below the wing of the atlas. For the neutral head and neck position, an auxiliary person held the horses with their heads straight in the sagittal plane. To achieve the extended position, the head of the horse was maximally distended forward. For the flexed position, side reins and a surcingle were used to position the ridge of the nose vertical to the floor or slightly behind the vertical. Laterolateral radiographs were obtained with the central beam targeted on the region of the larynx. Radiographs and lateral photographs of the horses were obtained at the same time for retrospective assessment of the head and neck positions.

The radiographs were evaluated using software for digital imaging (easyIMAGE; VetZ GmbH, Hannover, Germany). The pharyngeal diameter was defined as the shortest distance between the epiglottis and the roof of the pharynx (Figure 3). This distance and the length of the metallic markers were measured, and the amplification factor was determined as described by Cehak et al. [16] for calculation of the real pharyngeal diameter. For objective quantification of the head and neck positions, the GA and WA were measured as described above using the lateral photographs.

Data for all of the measured angles derived from pictures of the exercising horses were collated, and the means and standard deviations of the angles in the various head and neck positions were calculated and compared. Statistical analysis of the angles and pharyngeal diameters was performed with SPSS version 20 software (IBM, Armonk, NY, USA). Normal distributions of the collected data were confirmed by the Kolmogorov-Smirnov test and Q-Q plots. Because no linear connection was found, Spearman’s rank correlation coefficient was used to assess a possible paired correlation between the pharyngeal diameter and the GA or the WA. The significance threshold was set at p < 0.01.

## Results

The GA and the WA during exercise (GAe and WAe, respectively), measured on the photographs of the head and neck positions, which were rated subjectively beforehand, are shown in Table 1. For the unrestrained head and neck position—with no contact between the rider’s hand and the bit—the GAe was always <85° and the WAe >90°. The measured GAe in the head and neck position with neck elevated—the poll being the highest point and the ridge of the nose slightly in front of the vertical—varied between 60° and 90°, and the WAe was between 75° and 90°. For the head and neck position of hyperflexion—ridge of the nose behind the vertical—the measured GAe was always >90°, the WAe value was irrelevant.

**Table 1:** GAe and WAe in three head and neck positions in horses during exercise

**Head and neck position**	**Ground angle during exercise**	**Withers angle during exercise**
Unrestrained	< 85°	> 90°
Elevated neck	60°–90°	75°–90°
Hyperflexion	> 90°	Irrelevant

**Figure 4:**
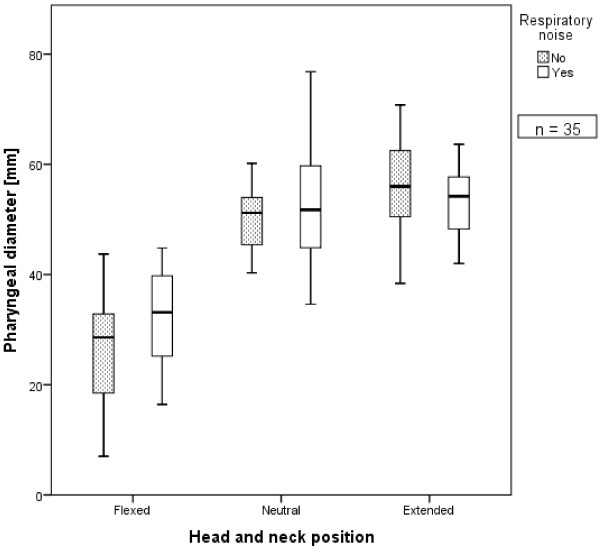
**Radiographic pharyngeal diameter as a function of the head and neck positions (flexed, neutral, extended).** There was a significant difference in the pharyngeal diameters between the flexed head and neck position and the neutral and extended head and neck positions. There was no significant difference between the pharyngeal diameters measured in the neutral and extended head and neck positions. There were no significant differences in pharyngeal diameter at each head and neck position between horses with and without respiratory noise during exercise. The boxes define the upper and lower quartiles with the medians marked by the horizontal lines. The whiskers indicate the minimum and maximum values (*n* = 35).

The calculated pharyngeal diameter, determined with the horses at rest, varied between 7.0 and 79.1 mm (Table 2). The smallest diameter (7.0 mm) occurred in the flexed head and neck position. The largest diameter (79.1 mm) was found in the extended head and neck position. In the flexed head and neck position, the pharyngeal diameter ranged from 7.0 to 44.8 mm (mean ± SD, 28.5 ± 9.6 mm). In the neutral head and neck position, it ranged from 34.6 to 76.8 mm (51.3 ± 8.87 mm). In the extended head and neck position, it ranged from 38.4 to 79.1 mm (55.6 ± 8.9 mm) (Table 2 and Figure 4).

**Table 2:** Ranges of pharyngeal diameter, GAr, and WAr in horses at rest

**Head and neck position**	**Pharyngeal diameter [mm]**	**Ground angle at rest [°]**	**Withers angle at rest [°]**
Flexed	28.47 ± 9.6	89.8 ± 6.11	90.3 ± 4.99
Neutral	51.26 ± 8.88	72.31 ± 6.73	109.54 ± 6.19
Extended	57.57 ± 8.92	50.87 ± 7.33	127.98 ± 9.38

As expected, the smallest ground angle at rest (GAr) (29.9°) was found in the extended head and neck position and the largest GAr (103.7°) in the flexed head and neck position. In contrast, the smallest WAr (78.7°) occurred in the flexed head and neck position and the largest WAr (144.7°) in the extended head and neck position.Of the 35 horses included in this study, 16 developed abnormal respiratory noise during the performance test. There were no significant differences between the determined pharyngeal diameters at rest in this group compared with those of the 19 horses without abnormal respiratory noise during exercise (Figure 4).

Among the 16 horses with abnormal respiratory noise during the performance test, two were referred for evaluation of poor performance, eight showed left-sided laryngeal dysfunction during an endoscopic examination at rest, and 13 were diagnosed with left-sided laryngeal dysfunction during an endoscopic examination at exercise. Apart from laryngeal dysfunction, three horses developed dorsal displacement of the soft palate (DDSP), and one horse experienced pharyngeal collapse during the endoscopic examination at exercise.

None of the 19 horses that did not display abnormal respiratory noise during exercise showed dysfunction of the upper airway during an endoscopic examination at rest. Six of these horses, however, developed left-sided laryngeal dysfunction during exercise, including one horse that had been referred for evaluation of poor performance. Another horse was diagnosed with axial deviation of the aryepiglottic fold, and one was diagnosed with DDSP.

**Figure 5:**
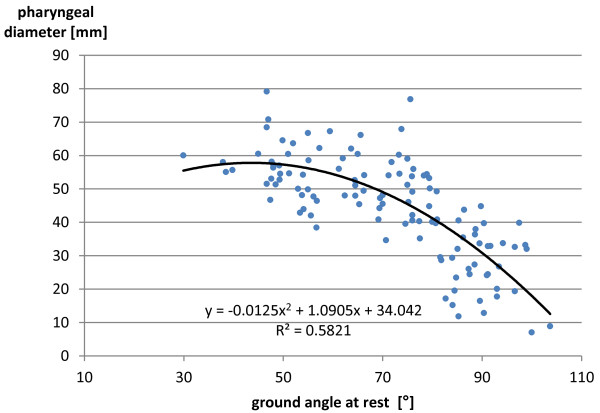
**Scatter diagram of the radiographic pharyngeal diameter as a function of the ground angle at rest.** There was a significant correlation between the radiographic pharyngeal diameter and the ground angle at rest (shown in Figure 1). With a polynomial trend line up to the power of two, R^2^ = 0.58 was achieved.

**Figure 6:**
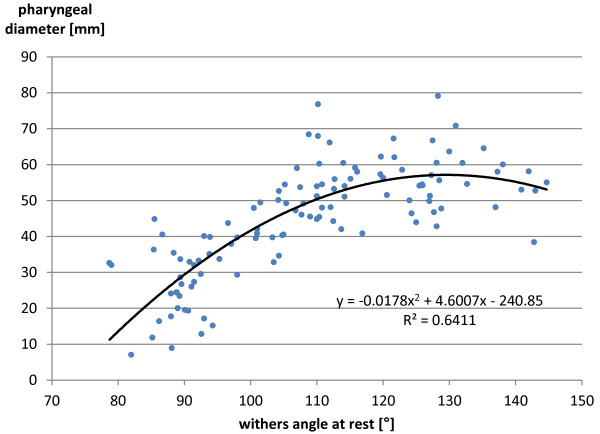
**Scatter diagram of the radiographic pharyngeal diameter as a function of the withers angle at rest.** There was a significant correlation between the radiographic pharyngeal diameter and the withers angle at rest (shown in Figure 2). With a polynomial trend line up to the power of two, R^2^ = 0.64 was achieved.

There was a significant difference in the pharyngeal diameters between the flexed head and neck position and the neutral and extended head and neck positions. No significant difference could be found between the pharyngeal diameters measured in the neutral and extended head and neck positions.Spearman’s rank correlation coefficient between the pharyngeal diameter and the GAr was −0.769 with p < 0.01. That between the pharyngeal diameter and the WAr was 0.774 with p < 0.01. Figures 5 and 6 represent scatter charts plotting one data marker for every pair of pharyngeal diameter and GAr and pharyngeal diameter and WAr, respectively. Polynomial trend lines up to a power of two were added to these scatter charts.

## Discussion

For objective interpretation of the head and neck position, two newly defined angles were established (GA and WA). These two angles were chosen to characterize atlanto-occipital flexion (WA) and cervicothoracic flexion (GA). As shown in Table 1, the combination of the WAe and the GAe provides an unambiguous method for judging the various head and neck positions in exercising horses.

The head and neck positions reviewed in the present study were named differently for the investigations at rest and those during exercise. The reason for this distinction is that a sedated horse at rest cannot have the same muscle tension as a horse in motion, and the contact between the bit in the horse’s mouth and the rider’s hands is missing. The head and neck position termed “elevated neck” indicates that the neck is raised, forming a harmonious curve from the withers to the poll (the highest point) with the head slightly in front of vertical. This position can be achieved only by a horse being ridden. When the head and neck position was “unrestrained”, most horses showed forward and slightly downward stretching—which could not be put on a level with the head and neck being maximally distended forward in a resting horse. Because of these unequal circumstances, the head and neck positions were named differently, and the ranges of angles in similar but not identical head and neck positions show slight differences.

During the exercise test all requested head and neck positions were achieved without using force. It should be noted, that the non-use of force was not determined by objective measurable parameters, but all requested head and neck positions were achieved without noticeable resistance against the riders aids and without the use of auxiliary reins. That and the various working disciplines and levels of training of the horses are the reasons why not all horses were able to perform all of the required head and neck positions. Hence, some horses had to be excluded from further analysis of one or more head and neck positions during exercise. Especially the head and neck position “hyperflexion” may have a negative connotation, not only because it has been subject of many studies but also the press discussed it extensively. In the current study the head and neck position “hyperflexion” was achieved, if the ridge of the nose was behind vertical. This should not be equated with the extreme overbending of the poll and neck –also called “Rollkur” [1]- which has been banned by the FEI [17,18] and could only be implemented by horses trained accordingly. The aim of the present study was to develop a method for categorizing head and neck positions objectively. In order to test this method it was not of importance which head and neck positions were investigated but rather that the selected head and neck positions could be differentiated unambiguously. As confirmed by the results of our study the two newly introduced angles are suitable tools describing head and neck position in an objective way. Therefore these angles may provide an opportunity to differentiate the various forms of hyperflexion, defined by the FEI [18], free from subjective bias.

No significant dependence of the pharyngeal diameter on the existence of a respiratory noise during exercise could be detected. The fact that the majority of horses showing an abnormal respiratory noise during exercise were diagnosed with laryngeal and not pharyngeal instability, could be an explanation.

In the second part of the current study, the GAr and the WAr were used to determine head and neck positions in resting horses. Three head and neck positions (neutral, extended, flexed) and the influence of each on the pharyngeal diameter in horses at rest were probed. There was a significant correlation between the pharyngeal diameter and the analyzed head and neck position and between the pharyngeal diameter and both the GAr and the WAr.

Figure 6 shows that increasing the WAr results in an increasing pharyngeal diameter. The maximum pharyngeal diameter is reached at approximately 115°. Beyond that point, despite continuing to increase the WAr, the pharyngeal diameter slightly decreased. This phenomenon occurs because hyperextension of the head and neck leads to greater hyperextension of the atlanto-occipital joint than of the atlantoaxial joint owing to the disparity in the range of motion of these two joints [19].

Radiographs were obtained from all of the sedated horses, regardless of the respiration phase. This method was chosen because an earlier study found no detectable relation between the pharyngeal diameter and the phase of respiration or the sedation status in horses at rest [16]. In the same study, a value of 29.6 ± 11.3 mm (mean ± SD) was published for the smallest pharyngeal diameter detected from the radiographs of healthy horses at rest. In the current study, the smallest measured pharyngeal diameter (7.0 mm) occurred in the flexed head and neck position in a healthy horse that did not develop abnormal respiratory noise during the exercise test. This minimum pharyngeal diameter appeared in combination with the maximum measured GAr of 103.7°, which implies that the ridge of the nose must be more than 10° behind vertical. In the studies mentioned before, flexion of the head and neck was performed only to the point where the ridge of the nose reaches vertical. Perhaps the higher flexion of the poll led to severe reduction of the pharyngeal diameter in this horse. Another possible explanation for this phenomenon is that the radiograph was obtained immediately after the head and neck of the horse was flexed—before the horse had swallowed to compensate for the positive pressure in the guttural pouches. The floor of the guttural pouches form the roof of the nasopharynx, and positive pressure within the guttural pouches could result in collapse of the roof of the nasopharynx [20]. It is also possible that the sedation status and the phase of respiration are more important than has been assumed. Some authors have postulated that the nasopharynx is more likely to collapse in a sedated horse than in a conscious horse because of the effect of sedation on muscular function. The major muscle preventing dorsal pharyngeal collapse is the stylopharyngeus muscle. Glossopharyngeal nerve anesthesia leads to stylopharyngeal dysfunction and consequently induces collapse of the dorsal pharyngeal walls [21]. Thus, sedation and the resulting relaxation of the stylopharyngeus muscle may lead to a similar effect. However, a previous study showed no statistically significant difference between the radiographic pharyngeal diameter determined with and without sedation in 10 horses [16].

In accordance with previous reports, the smallest pharyngeal diameter in resting horses was found in the flexed head and neck position [16,22]. Thus, the head and neck position in horses affects upper airway flow mechanics [2,9]. Decreasing the pharyngeal diameter leads to increased resistance in respiratory airflow. Particularly during exercise, when negative peak inspiratory airway pressures are reached, this may result in dynamic collapse of elements of the larynx or pharynx. Therefore, it is hypothesized that head and neck flexion is an important contributing factor for the development or exacerbation of dynamic collapse of the upper respiratory tract in horses during exercise [4,23-25]. Dynamic pharyngeal collapse and several cases of dynamic laryngeal collapse have been reported in association with head and neck flexion during exercise [10,26,27].

Further studies are needed to investigate the impact of objectively assessed head and neck positions on the upper airway tract during exercise.

## Conclusion

The aim of this study was to develop an objective, reproducible method for quantifying various head and neck positions accurately. Applying our new approach—a combination of the GA and WA —head and neck positions commonly used during training and competition in sport horses could be clearly measured and differentiated. In a previous study, an association between the pharyngeal diameter (determined on lateral radiographs of horses at rest) and the position of head and neck (identified subjectively) was proven. We succeeded in proving that the change in the pharyngeal diameter, measured as noted, is a function of the two newly introduced angles, which identify the head and neck position objectively.

Further studies will be performed to examine the influence of the various head and neck positions, assessed by the GA and WA, on upper airway function in exercising horses.

## Abbreviations

ADAF: Axial deviation of the aryepiglottic fold; DDSP: Dorsal displacement of the soft palate; FEI: Fédération Equestre Internationale; GA: Ground angle; GAe: Ground angle during exercise; GAr: Ground angle at rest; WA: Withers angle; WAe: Withers angle during exercise; WAr: Withers angle at rest.

## Competing interests

None of the authors has any financial or personal relationships that could inappropriately influence or bias the content of this article.

## Authors’ contributions

LG designed the study, carried out the radiographic examination, analyzed data, and drafted and wrote the manuscript. AKB contributed to the study design and helped draft the manuscript. BO contributed to the study design, data analysis, and interpretation. All authors read and approved the final manuscript.
